# Constipation and Lower Urinary Tract Dysfunction in Children and Adolescents: A Population-Based Study

**DOI:** 10.3389/fped.2016.00101

**Published:** 2016-10-03

**Authors:** Clara Sampaio, Ariane Sampaio Sousa, Luis Gustavo A. Fraga, Maria Luiza Veiga, José Murillo Bastos Netto, Ubirajara Barroso

**Affiliations:** ^1^Centro de Distúrbios Miccionais na Infância (CEDIMI), Escola Bahiana de Medicina e Saúde Pública, Universidade Federal da Bahia, Salvador, Brazil; ^2^Universidade Federal de Juiz de Fora, Juiz de Fora, Brazil

**Keywords:** constipation, incontinence, children, lower urinary tract dysfunction

## Abstract

**Objective:**

To evaluate the correlation between constipation and lower urinary tract dysfunction (LUTD) and nocturnal enuresis in a population-based study.

**Material and methods:**

This is a cross-sectional study. The criteria for inclusion were children and adolescents of between 5 and 17 years and who agreed to sign the informed consent form. The study excluded students with neurological problems or who had documented abnormalities of the urinary tract. To identify the presence and severity of LUTD, we used the Voiding Dysfunction Symptom Score (DVSS). To evaluate the presence of constipation, Rome III questionnaire was used.

**Results:**

We interviewed 829 children and adolescents, of which 416 (50.18%) were male. The mean (SD) age was 9.1 (±2.9) years. The overall prevalence of LUTD was 9.1%, predominantly in girls (15 versus 3.1%, *p* < 0.001). Constipation was found in 9.4% of boys and 12.4% of girls (*p* = 0.169). Constipated children were 6.8 times more likely to have LUTD than those not constipated (*p* < 0.001, coefficient and correlation of 0.411). Constipation was found in 8.2% of children without LUTD and in 35.2% of children with LUTD. We performed multivariate analysis to identify urinary symptoms that are independent predictors of the presence of constipation. The presence of infrequent urination (*p* = 0.004) and holding maneuvers (*p* < 0.001) were independent predictors. It was noted also noted that constipated children, according to the Rome III criteria, possess a worse DVSS (*p* < 0.001). Regarding the presence of nocturnal enuresis, 12.6% of children and adolescents had constipation in association with this symptom. However, this relationship was not statistically significant (*p* = 0.483).

**Conclusion:**

Constipated children were 6.8 times more likely to have LUTD than those not constipated. Among the urinary symptoms, infrequent voiding and holding maneuvers are independent factors of urinary expressions in constipated children. Children with more severe constipation have more prominent urinary symptoms. The presence of enuresis was not associated with constipation.

## Introduction

Constipation accounts for about 3–5% of consultations in pediatrics and up to 25% of consultations in pediatric gastroenterology (1). During childhood, one-third of children relate having had constipation at some time, most of whom had functional constipation (2). The presence of lower urinary tract dysfunction (LUTD) is a major cause of medical visits to pediatric urologists, accounting for up to 40% of cases (3). Its prevalence is around 2–25% of the population, varying with the intensity of symptoms measured (4).

The anatomical proximity between rectum and bladder and the innervation in common between the two structures may justify the association between constipation and LUTD (5), which is called bladder bowel dysfunction (BBD) (6). Children with BBD are more likely to develop emotional problems such as insecurity, anxiety, and low self-esteem, as well as behavioral problems (7).

Recently, Veiga et al. (8) demonstrated that constipation is associated not only with dysfunctional urination but also overactive bladder (OAB). In this study, 54.9% of children with OAB had constipation, in addition to being almost three times more likely to develop constipation than children without OAB. Some authors have demonstrated that children with dysfunctional voiding tend to have functional constipation, whereas children with OAB tend to have functional non-retentive fecal incontinence (9). Therefore, in approaching the child, it is essential that both disorders are investigated and treated.

The association between enuresis and constipation is also well established and known. In a study involving 30 enuretic children, Hogdes and Anthony (10) showed that 80% of them were also constipated and that these obtained significant resolution of their enuretic problem with laxative therapy. This association, however, needs further clarification preferably a population- or community-based study.

In spite of BBD being well described, to the best of our knowledge, there are no population-based studies to assess in detail the association between symptoms of constipation and lower urinary tract symptoms (LUTS). The term BBD is too generic: due to the large spectrum of LUTS presentation, the severity of urinary symptoms and constipation, different types of association between specific urinary and fecal symptoms could be found. In other words, constipation could be associated with more retentive symptoms such as low voiding frequency and children with a more severe constipation could present a more severe LUTS. Thus, this study aims to evaluate the correlation between constipation and LUTS and nocturnal enuresis in a population analysis.

## Materials and Methods

This is a cross-sectional study conducted in two Brazilian cities with a combined population of about 4 million. Data collection was carried out in public places from May to July 2015. The criteria for inclusion were children and adolescents of between 5 and 17 years who agreed to sign the informed consent form. Individuals who happened to pass by the collection points were approached at random and invited to participate. The study excluded individuals with neurological problems or who had documented abnormalities of the urinary tract. The study was submitted to the ethics committee of our institution and obtained approval for its implementation.

To identify the presence and severity of LUTD, we used the Voiding Dysfunction Symptom Score (DVSS), modified, and validated for the language in use (11, 12). This questionnaire contains 10 questions, of which 9 are related to clinical symptoms and 1 is related to environmental factors (social and family problems). For questions 1–9, numerical answers were taken, according to the Likert scale with scores from 0 to 3 based on the presence and severity of symptoms. For question 10, a score of 0 was given if there were no events related to stress or 3 if these were present. Girls with six or more points and boys nine or more points were considered to have LUTD.

The presence of urinary symptoms more than 1–2 times per week (score 1) was considered positive for each distinct question of the DVSS. Children who had urgency at least 1–2 times per week were considered to have an OAB. In order to investigate the presence of nocturnal enuresis, the children were asked whether or not they had experienced bedwetting events. The bedwetting episodes were subdivided into two categories: children who had less than three bedwetting events in a week and children who had more than three bedwetting episodes per week.

To evaluate the presence of constipation, we applied the Rome III child questionnaire, for ages 4–18 years. Participants who responded positively to two out of the six questions were considered constipated (13).

### Statistical Analysis

SPSS version 20.0 was used for statistical analysis. Numerical variables of age and score values were expressed as mean and SD or median and interquartile range (IQ). The variables included for analysis were age, gender, presence of enuresis, the DVSS, and Rome III scores. The age variable was divided into two groups: ≤9 and ≥10 years.

To perform the sample calculation, we estimated LUTD prevalence at 10% and accepted a difference of 3%, with 95% confidence level and requiring at least 385 participants in the survey.

Non-parametric variables of DVSS and Rome III scores were compared using the Spearman correlation, with *p* ≤ 0.05 considered significant. We performed a univariate analysis with chi-square test to compare proportions, with variables having *p* ≤ 0.05 considered significant, in order to test the association between constipation and the variables of nocturnal enuresis and LUTD. We adopted the manual procedure for the insertion and removal of variables.

## Results

We interviewed 829 children and adolescents, of which 416 (50.18%) were male. The mean (SD) age was 9.1 (±2.9) years, with a median of 9 (7–11) years.

The median score of the DVSS was 2 with IQ from 0 to 4 (range 0–18). According to the DVSS, the overall prevalence of LUTD was 9.1%, predominantly in girls (15 versus 3.1%, *p* < 0.001). Constipation was found in 9.4% of boys and 12.4% of girls (*p* = 0.169).

**Figure 1:**
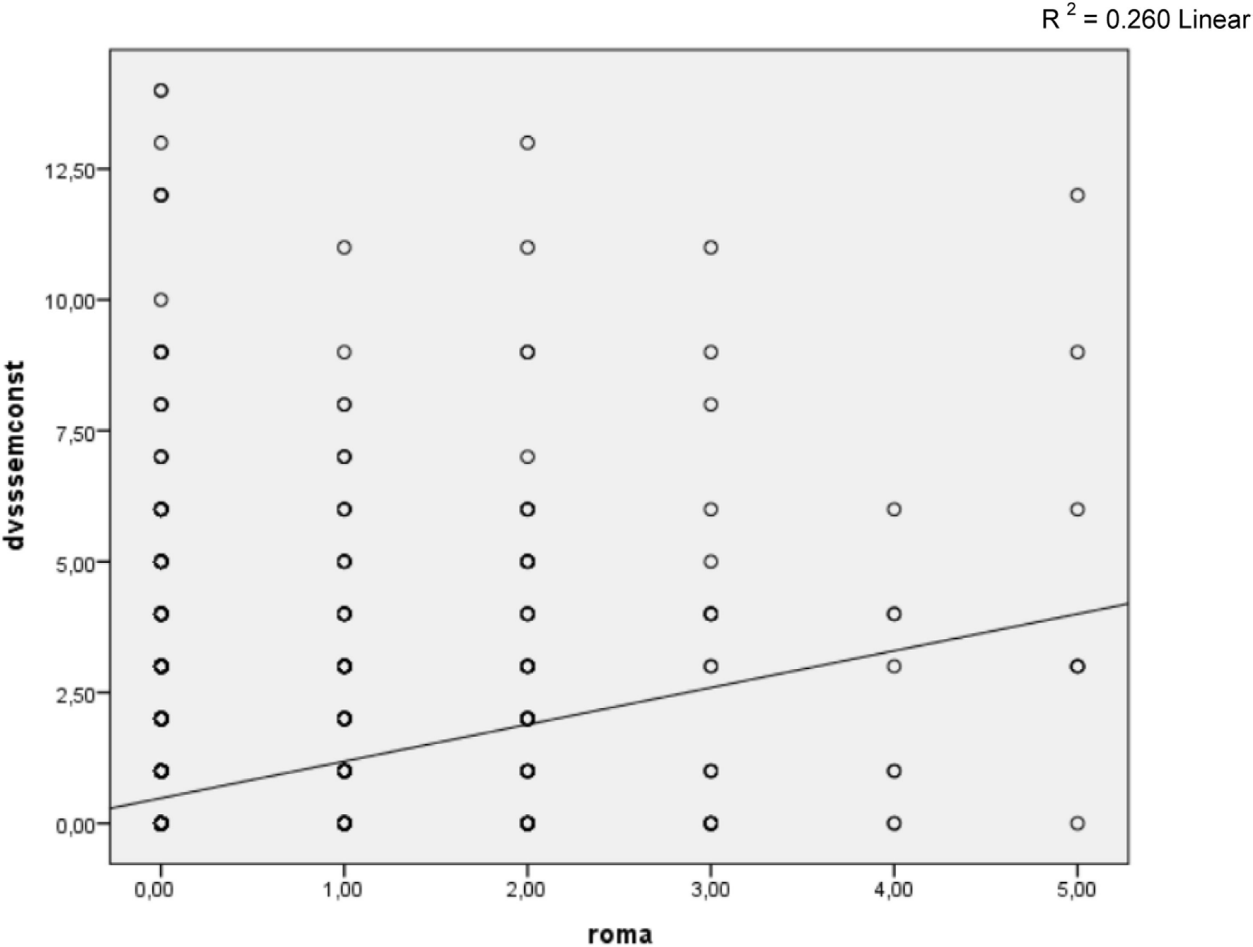
**Spearman correlation between DVSS score (disregarding questions related to intestinal rhythm) and Rome III criteria**. *R*^2^ = 0.260 linear.

A positive correlation was found between constipation and LUTD (Figure 1, *p* < 0.001, coefficient and correlation of 0.411). Constipated children were 6.8 times more likely to have LUTD than those not constipated (Table 1). Constipation was found in 8.2% of children without LUTD and in 35.2% of children with LUTD (*p* < 0.001, Table 1). However, 92.8% of non-constipated children did not have LUTD.

**Table 1:** **Relationship between constipation and the presence of LUTD**.

	Without LUTD	With LUTD	*p* Value	OR	CI (95%)
Not constipated	698 (92.8%)	38 (8.2%)	0.000	6.78	3.83–12.00
Constipated	65 (64.8%)	24 (35.2%)			
Total	763	62			

Table 2 shows the relationship between constipated and non-constipated children according to responses on urinary issues in the DVSS. It may be noted that urinary incontinence, infrequent voiding, holding maneuvers, urinary urgency, dysuria, and stressful event, respectively, were associated with the presence of constipation. We performed multivariate analysis (Table 3) to identify urinary symptoms that are independent predictors of the presence of constipation. The presence of infrequent urination (*p* = 0.004) and holding maneuvers (*p* = 0.000) were the only independent predictors.

**Table 2:** **Presence of urinary symptoms, according to DVSS in constipated and non-constipated children**.

	Without constipation	With constipation	Total	*p* Value	OR	CI (95%)
**Had they had wet underwear during the day?**						
No	636 (88.8%)	80 (11.2%)	716	0.629	0.849	0.437–1.649
Yes	103 (90.4%)	11 (9.6%)	114			
**When wet, is the underwear soaked?**						
No	688 (89.7%)	79 (10.3%)	767	0.033	2.049	1.048–4.007
Yes	51 (81%)	12 (19%)	63			
**How often do bathroom visits occur only 1 or 2 times in a day?**						
No	701 (90.6%)	73 (9.4%)	774	0.000	4.549	2.470–8.375
Yes	38 (67.9%)	18 (32.1%)	56			
**Does your child try to hold in the pee by crossing legs, squatting, or “dancing”?**						
No	561 (92.4%)	46 (7.6%)	607	0.000	3.083	1.977–4.807
Yes	178 (79.8%)	45 (20.2%)	223			
**When they have to pee, do they have to get to the bathroom quickly?**						
No	620 (90.1%)	68 (9.9%)	688	0.028	1.762	1.056–2.940
Yes	119 (83.8%)	23 (16.2%)	142			
**Do they need to push to pee?**						
No	733 (89.2%)	89 (10.8%)	822	0.202	2.745	0.546–13.808
Yes	6 (75%)	2 (25%)	8			
**Do they feel pain when they pee?**						
No	724 (89.8%)	82 (10.2%)	806	0.000	5.298	2.248–12.486
Yes	15 (62.5%)	9 (37.5%)	24			
**Have they experienced any stressful situation?**						
No	636 (90.1%)	70 (9.9%)	706	0.037	1.764	1.029–3.024
Yes	103 (83.7%)	20 (16.3%)	123			

**Table 3:** **Multivariate analysis of urinary symptoms present in constipated children**.

Variable	OR	*p*	CI (95%)
How often do bathroom visits occur only 1 or 2 times in a day?	1.577	0.004	(1.156–2.152)
Does your child try to hold in the pee by crossing legs, squatting, or “dancing”?	1.594	0.000	(1.274–1.994)
Constant	0.071	0.000	

*Multivariate analysis; LUTS, lower urinary tract symptoms*.

The final score of the DVSS, after eliminating questions about intestinal rhythm, which compares constipated and non-constipated children, is described in Table 4. It is noted that constipated children, according to the Rome III criteria, possess a worse DVSS (*p* < 0.001).

**Table 4:** **Final score obtained in DVSS, without constipation questions, for constipated and non-constipated children**.

DVSS points	Non-constipated	Constipated	Total	*p* Value
0	392 (53.0%)	21 (23.3%)	413 (100%)	0.000
1–4	277 (37.5%)	45 (50.0%)	322 (100%)	
5–8	54 (7.3%)	16 (17.8%)	70 (100%)	
9–14	16 (2.2%)	8 (8.9%)	24 (100%)	
Total	739 (100%)	90(100%)	829	

Regarding the presence of nocturnal enuresis, 12.6% of children and adolescents had constipation in association with this symptom (Table 5). However, this relationship was not statistically significant (*p* = 0.483). Still, 89.8% of non-constipated children were not enuretic, as well.

**Table 5:** **Correlation between constipation and nocturnal enuresis**.

	Without nocturnal enuresis	With nocturnal enuresis	*p* Value	OR	CI (95%)
Not constipated	651 (89.8%)	74 (10.2%)	0.483	1.27	0.647–2.504
Constipated	76 (87.4%)	11 (12.6%)			
Total	727	85			

## Discussion

Our study shows that LUTD is associated with constipation in children. Although this association is well reported in the literature, there are surprisingly few studies on this topic with population samples. In our sample, 27% of constipated children have LUTD, with a 6.782 times greater chance of having voiding dysfunction compared with those who are not constipated.

One study has shown that 6.3% of school-age children have daytime urinary incontinence (14) and that some risk factors may be attributed to daytime urinary incontinence such as encopresis, nocturnal enuresis, and social concerns (15). As for constipation, a systematic review study (16) has shown that the prevalence of constipation among children ranged between 0.7 and 29.6%, with a median of 12%. Another study has reported that 36% of Brazilian children in public schools are constipated, with no differences among boys and girls (17).

In order to determine the prevalence of dysfunctional voiding symptoms and abnormal bowel habits, Chung et al. (18) found in their study that the prevalence of both abnormal voiding and bowel habits is 46.4 and 31.3%, respectively, among Korean children on a nationwide basis. Curiously, 18.4% of Korean children had both abnormal voiding and bowel habits. Only, 5.6% of children had nocturnal enuresis.

Surprisingly, although the relationship between constipation and LUT dysfunction is well established and well known, few articles address this issue. Those who have addressed the issue have only used convenience samples and additionally have not used a Rome III criteria questionnaire to evaluate the degree of constipation.

The study by Hadjizadeh and colleagues (19) classifies a convenience sample of children with functional constipation in which modified DVSS is applied (without the questions related to constipation). It found that 63.5% of constipated children had voiding dysfunction according to the DVSS. However, Rome III criteria were not used to determine the presence of constipation in the children studied. Bolat and colleagues (20) conducted a population-based study in Turkey in order to identify risk factors associated with daytime urinary incontinence. They applied DVSS to obtain the information relating to urinary and bowel habits of children, noting that constipated children are 2.6 times more likely to have daytime urinary incontinence. In that study, the Rome III criteria were not used.

It is important to use the criteria of Rome III to diagnose constipation in children, given the objective of standardizing the definitions of gastrointestinal disorders such as constipation (21). The application of this criterion in population samples is observed in the literature (22, 23), and yet there are few studies employing the Rome III criteria in correlation studies with LUTD.

One important finding in our study, which to the best of our knowledge has not been reported in the literature, was that children with more severe constipation symptoms had more intense urinary symptoms. Koff et al. (6) showed that voiding and defecatory symptoms, such as infrequent urination and fecal retention, are associated with vesicoureteral reflux and urinary tract infection, suggesting a worse clinical picture when BBD is active. However, when diagnosing constipation and LUTD by using the Rome III and DVSS, as was done in our study, it is possible to measure the impact of constipation on LUTS.

Although the relationship between LUTD and constipation is well known, the most common urinary symptoms in constipated children were not well elucidated until now. BBD, according to ICCS, is defined as a combination of functional bladder and bowel disturbances (24). The definition, however, does not embrace the whole spectrum of LUT dysfunction. We know that LUT dysfunction presentation is diverse, and each symptom may suggest various meanings. Our study shows that symptoms which represent delaying of micturition such as holding maneuvers and reducing the number of micturitions per day are independently associated with constipation. The degree of continence required to control urination and defecation depends on the proper use of the striated musculature of adjacent structures as well as the perception of stimuli coming from both the bladder (25) and the rectum. The relationship between the concomitant establishment of LUTD and constipation can be explained by considering the innervation of the structures involved in urinary and digestive tracts of the sacral region. Once the bladder and rectum have a common embryological origin, the motor neurons responsible for the innervation of these structures come from the existing nerve endings in S2 and S4. Furthermore, the external sphincters, both anal and urethral, also have common innervation *via* the pudendal nerve (26). Other explanations for the emergence of BBD include contraction of the anal sphincter during defecatory urgency may cause contraction of the urethral sphincter and reflexively inhibit bladder function; the reverse can also happen with contraction of the urethral sphincter during urinary urgency, inhibiting the defecatory function; a rectum full of feces can itself alter bladder function (27).

Loening-Baucke (28) demonstrated in a study that the treatment of constipation can improve urinary symptoms and, therefore, constipated children with voiding dysfunction have fewer recurrent episodes of urinary tract infection. Moreover, it is known that events that affect the child’s life, such as stress, trauma, or change in routine, can be the cause of voluntary retention of feces, accounting for the relationship between constipation and stressful events (29). In our study, the association between stressful events and constipation is apparent.

Voluntary retention of feces may be present in children with LUTD given that, just as some children perform holding maneuvers to hold in urine, in a similar way, these children also end up prolonging the trip to the bathroom to evacuate. Voluntary retention of feces is a major cause of functional constipation and can be triggered by previous episodes of painful removal of hardened and large amounts of feces (30). According to Morais and Tahan (31), before the urge to defecate, the child contracts the external sphincter of the anus and the gluteal muscles in order to prevent the elimination of feces and to succeed in retaining them. Retention maneuvers can also be used in LUTD, especially in OAB, due to involuntary detrusor contractions that manifest in children as urinary urgency; therefore, the contraction of the urethral external sphincter can promote relaxation of the detrusor by relieving the symptoms of OAB (32), which cause children normally to adopt postures such as crossing legs and squatting in order to suppress or relieve the involuntary bladder contractions (33).

Thus, the feces are retained, assisted by voluntary restraining. They lose liquid while remaining in the rectum and over time become more desiccated, which complicates their evacuation and leads to a clogged toilet. Furthermore, evacuation of dry and hardened feces becomes more troublesome, which causes a portion of the fecal mass not to be expelled during defecation and remain in the rectal ampoule. Stagnant feces accumulate in the ampoule to the point of belatedly stimulating the defecation reflex, which inadvertently causes the child to present fecal incontinence events.

This study showed no association between nocturnal enuresis and constipation, as did the study of Sarici et al. (34) which did not observe a significant association between constipation and enuresis, present in 13.2% of enuretic children in the study.

There are some limitations of this study. We did not evaluate the constipation by digital rectal examination or by rectal diameter on ultrasound. Also, we did not evaluate the dietary habits that could have some influence in the intestinal rhythm. Furthermore, this is a cross-sectional study and, therefore, we cannot establish any causal relationship.

## Conclusion

Constipated children were 6.8 times more likely to have LUTD than those not constipated. Among the urinary symptoms, infrequent voiding and holding maneuvers are independent factors of urinary expressions in constipated children. Children with more severe constipation have more prominent urinary symptoms. The presence of enuresis was not associated with constipation.

## Author Contributions

All authors listed have made substantial, direct, and intellectual contribution to the work and approved it for publication.

## Conflict of Interest Statement

The authors declare that the research was conducted in the absence of any commercial or financial relationships that could be construed as a potential conflict of interest.
